# Antibody-Targeted Imaging of Gastric Cancer

**DOI:** 10.3390/molecules25204621

**Published:** 2020-10-11

**Authors:** Komal Mandleywala, Shayla Shmuel, Patricia M. R. Pereira, Jason S. Lewis

**Affiliations:** 1 Department of Radiology, Memorial Sloan Kettering Cancer Center, New York, NY 10065, USA; mandleyk@mskcc.org (K.M.); shaylaofnyc@gmail.com (S.S.); 2 Molecular Pharmacology Program, Memorial Sloan Kettering Cancer Center, New York, NY 10065, USA; 3 Department of Pharmacology, Weill Cornell Medical College, New York, NY 10065, USA; 4 Department of Radiology, Weill Cornell Medical College, New York, NY 10065, USA; 5 Radiochemistry and Molecular Imaging Probes Core, Memorial Sloan Kettering Cancer Center, New York, NY 10065, USA

**Keywords:** molecular imaging, gastric cancer, PET, SPECT, optical imaging

## Abstract

The specificity of antibodies for antigens overexpressed or uniquely expressed in tumor cells makes them ideal candidates in the development of bioconjugates for tumor imaging. Molecular imaging can aid clinicians in the diagnosis of gastric tumors and in selecting patients for therapies targeting receptors with a heterogeneous intratumoral or intertumoral expression. Antibodies labeled with an imaging radiometal can be used to detect primary tumors and metastases using whole-body positron emission tomography (PET) or single photon emission computed tomography (SPECT), both during diagnosis and monitoring disease response. Conjugated with fluorescent dyes, antibodies can image tumors by targeted optical imaging. This review provides an overview of the most recent advances in the use of antibodies labeled with radiometals or conjugated with fluorescent dyes for gastric cancer imaging.

## 1. Introduction

Gastric (stomach) cancer is a global health concern: in 2018, more than 1,000,000 new cases were diagnosed and 783,000 deaths were registered worldwide [1]. Gastric cancer (GC) is usually diagnosed in its later stages [2] because of the late onset of symptoms and lack of standard or routine screening tests, resulting in a poor prognosis and high mortality rate [2].

Molecular analyses of gastric tumors include methods to test changes in the genes (e.g., gene amplification assessed using in situ hybridization, ISH) and/or in the expression of proteins, mostly via immunohistochemistry (IHC) [3]. In this context, detection of human epidermal growth factor receptor 2 (HER2) in GC often includes methods to determine *ERBB2* gene amplification and HER2 protein expression [4]. However, these different methodologies do not always give similar results and can be misleading when defining patient selection for anti-HER2 systemic therapy. Unfortunately, gastric cancer is a malignancy with high heterogeneity, at least in the setting of HER2 status. Determining HER2 status through multiple biopsies of the same patient could reduce the false- negatives and false-positives observed in GC [3]. Whole-body molecular imaging is also a powerful technique to be used in complement to IHS and IHC, as it allows the visualization of primary tumors and metastases in the same patient [5,6].

Tumor cells often have upregulated glucose transporters (GLUT). Fluorodeoxyglucose (FDG) positron emission tomography–computed tomography (PET-CT) has improved the staging of GC by combining functional (PET) and anatomical (CT) imaging to visualize tumor areas with high metabolic activity [7,8]. However, not all tumor lesions are avid for FDG and non-tumor cells also express GLUTs [7,8]. The use of FDG-PET is associated with false-negative and false-positive images that could misdirect therapy planning and decrease diagnostic accuracy. In this context, PET has evolved into immunoPET, wherein antibodies with high specificity for antigens overexpressed or uniquely expressed in tumor cells are labeled with PET radiometals [5,9,10,11,12,13,14]. In addition to PET, antibodies radiolabeled with single photon emission computed tomography (SPECT) radiometals allow noninvasive, highly sensitive imaging of GC [15,16]. Another attractive antibody-based imaging strategy utilizes comparatively innocuous fluorescent imaging probes that when conjugated to antibodies can be directed specifically to tumor-associated antigens and visualized with high tumor-to-background ratios [17,18,19,20].

In sum, antibodies labeled with PET/SPECT radiometals or fluorescent dyes allow for visualization of specific antigens present in gastric tumors or metastases—a vital component of diagnosis that also localizes the primary lesion to inform treatment options and allows clinicians to monitor disease progression. This review will focus on full-length antibodies labeled with PET radiometals, SPECT radiometals, and fluorescent dyes that have been used preclinically and clinically to image gastric tumors.

## 2. ImmunoPET and ImmunoSPECT with Full-Length Antibodies in GC

ImmunoPET and immunoSPECT are imaging techniques that use antibody-based radiotracers. ImmunoPET and immunoSPECT have been used for the non-invasive imaging of gastric cancer in both preclinical and clinical studies. The first section of the review will discuss the use of immunoPET in GC targeting the antigens carcinoma-associated antigen (MG7) [14], programmed death-1 (PD-1) [16], cadherin-17 (CDH17) [15], human epidermal growth factor receptors 2 and 3 (HER2 [5,9,21,22,23,24] and HER3 [12]), hepatocyte growth factor (HGF [11]), and the mesenchymal-epithelial transition factor (MET) [10]. As shown in Table 1, FDA-approved or newly developed antibodies targeting membrane antigens were radiolabeled with gallium-68 (^68^Ga), technetium-99m (^99m^Tc), indium-111 (^111^In), copper-64 (^64^Cu), zirconium-89 (^89^Zr), and bromine-76 (^76^Br) and used for PET or SPECT imaging of gastric tumors.

The following sections describe preclinical and clinical studies using radiolabeled antibodies and PET/SPECT imaging for the diagnosis of GC and monitoring receptor status during treatment.

### 2.1. MG7

MG7 is a gastric-cancer-specific antigen whose expression is positively correlated with disease progression [25]. MG7 is overexpressed in GC tissues when compared with normal mucosa or benign lesions and is present in over 90% of GC patients, suggesting its potential as a biomarker of the disease [25]. Initial studies of MG7-targeted immunoPET used the anti-MG7 antibody conjugated with the chelator 1,4,7-triazacyclononane-*N*,*N*’,*N*’’-triacetic acid (NOTA) [14]. The NOTA-MG7 conjugate was then radiolabeled with the short-lived radioisotope gallium-68 and used as a probe for in vivo imaging of BGC-823 gastric xenografts. The use of NOTA as a bifunctional chelator to radiolabel biomolecules with gallium-68 allows efficient labeling at room temperature, which is important to preserve the antibody’s immunoreactivity. The ^68^Ga-labeled immunoconjugate demonstrated a tumor uptake of 2.53 ± 0.28% ID/g at 60 min after intravenous (tail vein) injection of ^68^Ga-NOTA-MG7. In addition to the tumor, ^68^Ga-NOTA-MG7 accumulated in the liver and kidneys, probably due to the metabolism of the probe through these organs. Upon systemic administration, antibodies exhibit a relatively long biological half-life and slow biodistribution profile. For this reason, they are often labeled with longer-lived radiometals such as zirconium-89 (half-life of 3.3 days). Since gallium-68 has a relatively short half-life (67.7 min), a higher tumor-to-background ratio could be obtained by labeling the antibody with a longer-lived radioisotope. Another limitation on the use of ^68^Ga-NOTA-MG7 relates to the high expression of MG7 in *Helicobacter pylori*-associated gastric diseases, which could confound immunoPET results in patients [25].

### 2.2. PD-1

**Figure 1:**
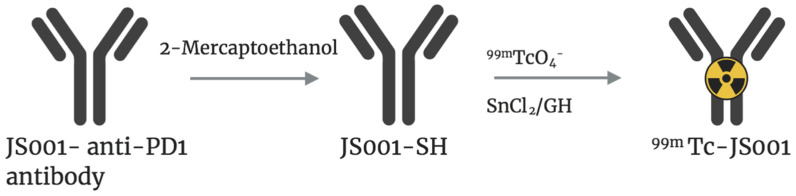
JS001-SH was obtained after reaction of the antibody with 2-mercaptoethanol. Direct radiolabeling of JS001-SH (as shown above) was achieved by reacting JS001-SH with Na[^99m^Tc0_4_] and SnCl_2_ in PBS buffer. Adapted from [16].

PD-1 is a T-cell co-receptor (with two ligands: PD-L1 and PD-L2) used in cancer immunotherapy [26]. PD-1 significantly reduces the patient’s immune system by de-activating the normal T-cell activity [27]. Currently, PD-1 expression is determined by IHC of tumor biopsies; however, this method is invasive and can only be performed from tumor tissue biopsies. Another downside is that PD-1 status can only be tested for the collected tissue, not for the whole tumor or other tumor lesions in the body. As such, antibody-targeted imaging combined with IHC could help detect PD-1 protein levels. A recent study by Guo et al. analyzed the use of ^99m^Tc-labeled JS001 as an immuno-SPECT tracer in an orthotopic BCG-823 GC mouse model [16]. JS001, a monoclonal antibody that targets PD-1, was reacted with 2-mercapthoethanol, affording JS001-SH. ^99m^Tc-JS001 was then obtained after mixing JS001-SH with glucoheptonic acid (GH), tin(II) chloride (SnCl_2_), and Na[^9 9m^TcO4] (Figure 1). While this immunoSPECT tracer exhibited distribution in the blood, liver, and kidneys as visualized in SPECT images, the conjugate also demonstrated the highest uptake in tumor cells relative to the adjacent stomach. However, a more quantitative study will be necessary to determine tumor-to-stomach ratios in order to define its potential utility as a PD-1 tracer in vivo.

### 2.3. CDH17

CDH17 is a transmembrane protein that functions as a peptide transporter and mediates cell-to-cell adhesion [28]. This protein plays a key role during embryo development and is found in the fetal liver and gastrointestinal tract [29]. In a healthy adult, the gene that codes for the CDH17 protein is silenced; however, it is often expressed in gastric cancer, as well as other adenocarcinomas. In a recent study, the potential of CDH17 as a biomarker in GC was investigated through imaging techniques [15]. The anti-CDH17 monoclonal antibody D2101 was radiolabeled with indium-111 and administered to mice with AGS gastric xenografts. SPECT/CT imaging demonstrated high uptake of ^111^In-labeled D2101 in tumors starting 24 h after the radioconjugate was administered and low uptake in surrounding organs. Images with high tumor uptake and low background level were obtained at 96 h after antibody administration. In ELISA assays, D2101 showed high specificity to CDH17-expressing gastric cancer cells. D2101 shown binding to human CDH17 but not murine CDH17, as observed by surface plasmon resonance analysis. Biodistribution and SPECT/CT images demonstrated that in addition to accumulating in the tumor, the radiolabeled D2101 binds other CDH17-positive organs such as the intestine, which may hinder its ability to detect metastases in lymph nodes around the stomach.

### 2.4. HER2

HER2 is a transmembrane tyrosine kinase receptor that is part of the epidermal growth factor receptor (EGFR) family. The EGFR family consists of EGFR, HER2, HER3, and HER4 [30]. High levels of the HER2 protein are common in gastric cancer, observed in nearly 30% of patients [31] and the anti-HER2 antibody trastuzumab is FDA-approved for gastric cancer therapy. The diagnosis of HER2 status is a prerequisite to initiate HER2-targeted therapies. The techniques for assessing HER2 overexpression include IHC and fluorescence in situ hybridization (FISH), both of which are invasive and risk causing metastasis during the procedures [3,32]. These methods can only be performed ex vivo, which may not reflect HER2 status in the entire tumor tissue and can make it difficult to differentiate between primary lesions and metastases. Indeed, HER2 heterogeneity exists not only between primary tumors and metastases, but also in the primary tumor alone, and within the same lesion before and after treatment [6]. In this context, PET and SPECT using radiolabeled anti-HER2 antibodies have demonstrated great potential to non-invasively image and monitor HER2 expression in both preclinical models and patients with GC [5,9,21,22,23,24].

HER2-directed PET imaging using radiolabeled trastuzumab has great potential to non-invasively visualize HER2-expressing cancer cells and monitor therapeutic target engagement in patients over a prolonged period [5,6,22]. ^89^Zr-labeled trastuzumab has shown success in delineating HER2-positive gastric cancer [5,6] and monitoring tumors’ response to HER2-directed therapies [22]. The first study evaluating ^89^Zr-labeled trastuzumab as an imaging agent in patients with HER2-expressing GC was reported in 2018 by O’Donoghue et al. [5]. ^89^Zr-trastuzumab biodistribution, pharmacokinetics, and dosimetry were evaluated in 10 patients, confirming HER2 status through immunohistochemistry or FISH [5]. PET imaging showed optimal tumor visualization 5–8 days after the antibody’s injection, with no significant toxicities observed and minimal uptake in non-tumor organs. These results show that the use of ^89^Zr-trastuzumab PET to detect HER2 status in GC is safe and effective. Other preclinical studies demonstrated that ^89^Zr-trastuzumab is more specific than ^18^F-FDG in imaging HER2-expressing gastric tumors [22].

Another important biological characteristic of the HER2 antigen relates to its internalization from the cell membrane to the intracellular compartment through an endocytic process. HER2 internalization reduces HER2 availability at the cell membrane and, consequently, decreases anti-HER2 antibody-tumor binding. Pereira et al. used cholesterol-depleting drugs in an attempt to temporarily stabilize HER2 at the cell membrane and enhance the ability of the ^89^Zr-labeled anti-HER2 antibodies trastuzumab [23] and pertuzumab [24] to bind to HER2-expressing gastric xenografts.

In addition to zirconium-89, trastuzumab was radiolabeled with the shorter-lived radioisotope copper-64 [9]. At 36 h after intravenous injection of the radioconjugate in PDX GC models, ^64^Cu-NOTA-trastuzumab demonstrated higher tumor uptake than the negative control ^64^Cu-NOTA-hIgG1. Additionally, radiolabeled trastuzumab accumulation was higher in HER2-expressing PDXs than in tumors containing low levels of the protein. Clinical studies further validated the ability of ^64^Cu-NOTA-trastuzumab to image GC [9].

Others have used molecular imaging during development of new anti-HER2 antibodies [21]. A study performed in 2018 by Kuo et al. attempted to test the tumor uptake of indium-111 labeled antibodies in NCIN87 gastric cancer cells [21]. The four anti-HER2 antibodies tested were H32 IgG, 75 IgG, 61 IgG, and trastuzumab. When comparing the four different monoclonal antibodies, ^111^In-labeled 61 IgG showed the highest tumor uptake in microSPECT imaging and biodistribution studies. Additionally, the radiolabeled 61 IgG demonstrated a high internalization rate when compared with H32 IgG, 75 IgG, or trastuzumab. Although the 61 IgG antibody showed positive results, there has been no follow-up preclinical research using the radioconjugate.

**Figure 2:**
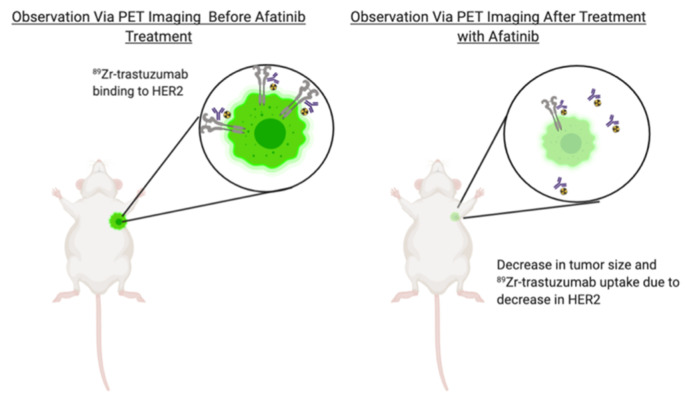
The figure above represents a mouse model with a NCNI-N87 gastric tumor that has undergone treatment with afatinib. Through ^89^Zr-labeled PET imaging, a decrease in tumor size and HER2 expression was observed after afatibinib treatment. The decrease in HER2 protein levels resulted in a decrease of ^89^Zr-trastuzumab accumulation in tumors and an increase of the radiotracer in the bloodstream [22].

Because HER2 levels can change during GC therapy, it is essential to identify alterations in receptor expression to properly adjust treatments. ImmunoPET plays a vital role in the monitoring of protein levels: by identifying antibody tumor uptake, one can determine changes in receptor levels. Previous studies by Janjigian et al. used ^89^Zr-trastuzumab to image HER2-positive GC before therapy with afatinib, i.e., an inhibitor of EGFR [22]. The results showed that tumor response to afatinib correlated with immunoPET, tumor reduction, apoptosis, and downregulation of HER2. Mice treated with afatinib showed a decrease in ^89^Zr-trastuzumab tumor uptake, which is a direct result of the decrease in HER2 expression in the tumors (Figure 2). In a clinical follow-up study, Sanchez-Vega et al. compared ^89^Zr-labeled trastuzumab accumulation in GC with patient response to afatinib; while trastuzumab demonstrated a homogeneous accumulation in tumors with high sensitivity to afatinib, tumors with heterogenous accumulation had a poor response [6].

Similar to afatinib, lapatinib is an irreversible tyrosine kinase inhibitor [13,33]. Lapatinib increases HER3 protein levels in HER2-expressing breast cancer, which may reduce the effect of the cancer drug therapy [34]. Therefore, antibody-PET targeting HER3 was used to evaluate HER3 status in tumors treated with lapatinib [12]. NCIN87 gastric xenografts that received lapatinib were administered ^89^Zr-labeled mAb3481 (anti-HER3) to image HER3 expression. In vitro studies showed that HER3 was internalized due to lapatinib, which also caused internalization of ^89^Zr-labeled mAb3481. While there was an observable increase in HER3 expression in vitro, lapatinib did not change HER3 expression in tumor xenografts. As a result, ex vivo results showed no change in HER3 expression with lapatinib treatment and, therefore, ^89^Zr-labeled mAb348 tumor uptake was similar in control versus lapatinib-treated tumors.

### 2.5. HGF/MET

HGF is a heterodimer protein that is present in healthy mesenchymal cells [35]. HGF is overexpressed in gastric cancer, as well as many other cancers. HGF is the ligand for the hepatocyte growth factor receptor (MET) [36]. MET is normally expressed in epithelial cells and is a tyrosine kinase protein [37]. Activation of MET by HGF leads to tumorigenic properties including increased cell scattering, migration, invasion, and proliferation. Preclinical studies have attempted to use HGF- and MET-targeted antibodies as imaging agents in GC [10,11].

HGF is inhibited with the HGF monoclonal antibody rilotumumab (AMG102) [38]. Preclinical studies demonstrated that ^89^Zr-DFO-AMG102 can noninvasively visualize HGF overexpression in murine xenografts [11]. ^89^Zr-DFO-AMG102 demonstrated high accumulation in HGF-expressing gastric xenografts and PDXs when compared with low-expressing GC.

Another study demonstrated that onartuzumab, an anti-MET humanized 1-armed monoclonal antibody from the family of IgG1 monoclonal antibodies [39], radiolabeled with bromine-76 or zirconium-89, has the potential to visualize MET expression in gastric tumors [10]. When compared with ^76^Br-labeled antibody, ^89^Zr-labeled onartuzumab demonstrated high tumor accumulation and tumor-to-muscle ratios [10].

## 3. Antibody-Fluorescent Dyes for GC Diagnosis

Endoscopic procedures are used to screen high risk symptomatic patients by detecting morphological changes in gastrointestinal mucosa. Even as a gold standard technique, endoscopic procedures do not allow effective differentiation of neoplastic gastric lesions from healthy or stressed tissue due to their indiscernible microstructural differences [40]. Nam et al. have demonstrated that early detection of GC requires repeated endoscopic procedures owing to the rate of false negatives associated with the procedure [41]. While conventional endoscopy with white-light imaging provides information related to morphological irregularities, it can still be challenging to demarcate boundaries between pre-malignant lesions and healthy mucosa. Hence, gastric tumors are often incompletely resected, which can lead to a higher recurrence rate and ultimately, poor prognosis [42]. The introduction of the novel techniques chromo- and magnifying- endoscopy with narrow-band imaging has enhanced accuracy in identifying GC [43,44,45,46].

**Figure 3:**
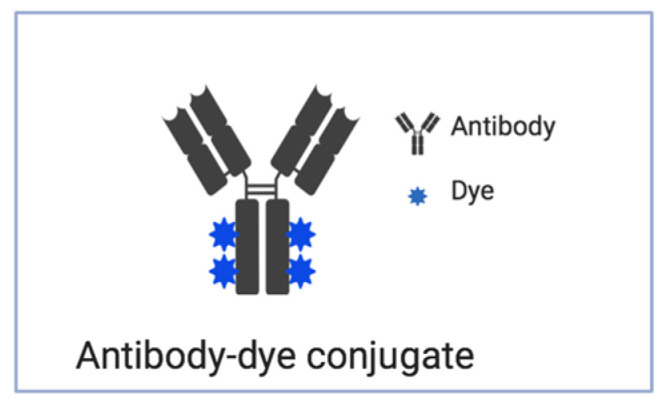
Schematic of possible antibody-dye conjugates obtained after labeling an antibody with dyes.

Optical imaging technologies such as bioluminescence and fluorescence have been increasingly explored over the last decade as an alternative, able to overcome the visual limitations of conventional white-light endoscopy. These cost-effective and minimally invasive optical imaging modalities rely on the light-emitting properties of individual dyes. Antibodies conjugated with such dyes have proven to be excellent tools for targeted optical imaging. Additionally, compounds classified as photosensitizers, upon light activation, serve both diagnosis and therapeutic purposes in photodynamic therapy [47,48,49]. The next sections will briefly describe dyes conjugated with antibodies for GC imaging (Figure 3, Table 2).

Fluorescent Endoscopy.

While fluorescence imaging has shown positive results, the diagnostic accuracy of this technique is limited by the reduced selectivity to tumor cells of its fluorescent dyes—fluorescein, methylene blue, and indocyanine green (ICG). These are clinically available vascular contrast agents that allow detection of superficial alterations in microvasculature and structural morphology (instead of molecular abnormalities) and require additional confirmation through histopathological analysis of a biopsy sample [50]. However, biopsies are invasive, expensive, and cause bleeding complications. To overcome these practical challenges, combinations of multiple modalities that leverage critical qualities of each technique seem intuitively appealing.

**Figure 4:**
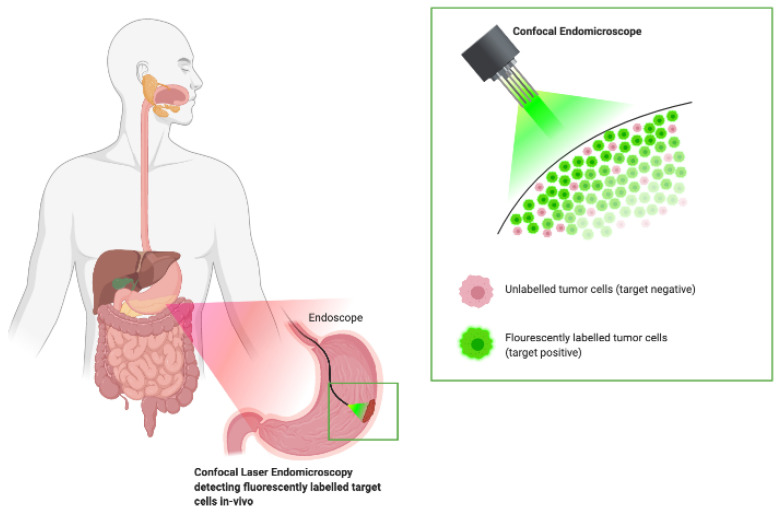
Schematic representation of fluorescent antibody-based confocal laser endomicroscopy for real-time visualization of the extent of the tumor as well as molecular characterization of a suspected neoplastic lesion. Antibodies conjugated with fluorophores (fluorescein, Alexa-flour488, FITC) bind to membrane-associated target proteins expressed in gastric neoplastic tissue (shown in green). The confocal endomicroscope directs light of a specific wavelength (488 nm), thus exciting the fluorochrome and allowing tumor visualization. Confocal images are captured by the endoscope, facilitating in vivo analysis to select patients based on antigen expression patterns.

“Red-flagging” tumor-associated antigens with fluorescently labeled antibodies may provide improved specificity and sensitivity compared to currently available diagnostic techniques. In this context, confocal laser endomicroscopy (CLE) uses in vivo microscopy during an ongoing endoscopy to magnify the microstructural aberrations for enhanced visualization [51]. The application of antibody-based fluorescent dyes (administered either systemically or topically) in combination with a CLE system may provide a reliable platform for biomarker-guided endoscopy and, subsequently, individualized therapy [52] (Figure 4). Initial studies have been performed with promising results and, as the technology evolves, such combinations of strategies may allow real-time targeted optical screening for GC in suspected patients and assess the molecular status of cancerous lesions during ongoing therapy.

### 3.1. EGFR-Targeted Fluorescent Imaging

Epidermal growth factor receptor (EGFR) is overexpressed in GC; high EGFR protein levels are associated with advanced GC and poor prognosis [53]. For assessment of molecular fluorescence imaging, preclinical studies used the anti-EGFR antibody cetuximab labeled with fluorescein isothiocyanate (FITC) to image EGFR-expressing MKN45 cancer cells [17]. Molecular in vivo imaging was performed using a CLE system; strong fluorescence intensity was observed in 7 out of 9 mice with tumors, whereas no specific fluorescence was observed in the control group. Additionally, a fluorescence pattern corresponding to receptor-mediated endocytosis was observed in the tumor tissue. This theranostic approach of using fluorescent-labeled cetuximab as a probe confers the advantage of simultaneously delivering a targeted treatment and measuring therapy response through imaging.

### 3.2. MG7-Targeted Fluorescent Imaging

As described in the previous section, MG7 is highly expressed in poorly differentiated gastric carcinoma [25]. Li et al. demonstrated the feasibility of using Alexa Fluor 488-labeled anti-MG7 antibody in BGC-823 and SGC-7901 xenograft models of human GC [19]. A Five1 scan handheld endoscopic instrument was used, and maximum tumor fluorescence intensity was observed at 48 h after intracardiac administration of the AF488-antibody conjugate. An approximate quantification of MG7 antigen distribution could be achieved by fluorescently tagging MG7 antibodies. Such an approach can be extrapolated to achieve real-time analysis of intrinsic MG7-antigen expression in order to screen suspected patients and predict therapeutic response. In addition, a semi-quantitative assessment of MG7 protein levels was performed on CLE images obtained after incubating fresh ex vivo surgical and human tumor biopsies in AF488-labeled MG7. The results were compared to corresponding IHC data and a significant correlation was established between varying grades of expression level. However, further quantitative investigations are required for accurate evaluation.

### 3.3. VEGF-Targeted Fluorescent Imaging

The VEGF signaling pathway plays a pivotal role in promoting angiogenesis in endothelial cells [54]. High VEGF expression has been shown to stimulate the growth of tumor-associated vasculature and metastases [55]. Therefore, preclinical studies aimed at developing strategies for gastric cancer diagnosis have used fluorescent antibodies targeting VEGF. Previous studies by Foersch et al. demonstrated the implementation of the CLE system with a fluorescently labeled anti-VEGF antibody to visually target VEGF in both xenografts (SW480/SW620) and genetic mouse models (APCmin) of gastrointestinal cancer [20]. Moreover, using intravenously administered AF488-labeled antibody targeting VEGF, transition zones from healthy tissue to tumors could also be distinguished, which could be particularly useful in facilitating lesion margin delineation and serve as a guide for surgical resection. A limiting factor in this study is that in some cases, increased VEGF expression might result from inflammatory conditions [56], generating misleading false-positive results.

## 4. Conclusions

Treatments for all cancers are specific to the primary tumor lesion as well as the specific antigens that may be present in the unique tumor. Most common cancer therapies are in the form of chemotherapy, radiation therapy, or surgery. GC therapies include surgery, chemotherapy, targeted therapies, immunotherapy, or radiation therapy. The type of treatment depends on the stage of the disease and the biomolecular features of the tumors. The results obtained from IHC and ISH can be misleading when selecting patients for targeted therapies. In addition to the infeasibility of collecting all the neoplastic lesions that might be present in a patient at the time of diagnosis, information obtained from biopsies is not an accurate representation of the entire tumor. Gastric cancer is a heterogeneous disease—not only when comparing a primary lesion with metastases, but also intratumorally.

Antibody-based imaging strategies specific for certain antigen-overexpressing GC allow the visualization of primary tumors and metastases with high contrast. In this context, antibody-PET and antibody-SPECT have the potential advantage of noninvasively determining changes in antigen (e.g., HER2) expression and target engagement in both the primary tumor and metastases. A novel integration of fluorescence-tagged antibodies and confocal laser endoscopy for prompt visualization of the dynamic molecular footprint also represents a promising pathway towards individualized therapy.

## Figures and Tables

**Table 1:** Radiolabeled antibodies used in molecular imaging of gastric tumors.

Biological Model	Target	Antibody	Radioisotope	Main Findings	Reference
BGC-823 subcutaneous xenografts	MG7	MG7	^68^Ga	Accumulation in the tumor, liver, and kidneys.	[14]
BCG-823 orthotopic tumors	PD-1	JS001	^99m^Tc	Accumulation in the tumor, blood, liver, and kidneys.	[16]
AGS subcutaneous xenografts	CDH17	D2101	^111^In	Optimal tumor accumulation was achieved at 96 h after ^111^In-DS2101 administration.	[15]
NCIN87 subcutaneous xenografts	HER2	H32 IgG, 75 IgG, 61 IgG, and trastuzumab	^111^In	^111^In-labeled 61 IgG showed the highest tumor accumulation.	[21]
Patient-derived gastric xenografts and patients		trastuzumab	^64^Cu	The combination of ^64^Cu-NOTA with trastuzumab showed higher tumor uptake than trastuzumab alone.	[9]
Patients with HER2-expressing gastric tumors		trastuzumab	^89^Zr	Tumor accumulation showed optimal results at 5-8 days after ^89^Zr-trastuzumab injection in patients.	[5]
NCIN87 subcutaneous xenografts		trastuzumab	^89^Zr	Afatinib downregulated HER2 protein levels and reduced tumor size.	[22]
NCIN87 subcutaneous xenografts		trastuzumab pertuzumab	^89^Zr	The endocytic protein caveolin-1 affects trastuzumab and pertuzumab binding to HER2-expressing gastric tumors.	[23,24]
NCIN87 subcutaneous xenografts	HER3	mAb3481	^89^Zr	Lapatinib treatment resulted in internalization of HER3 and ^89^Zr-mAb3481.	[12]
NCIN87 subcutaneous xenografts	HGF	AMG102	^89^Zr	^89^ZrDFO-AMG102 is an effective antibody for determining HGF expression in murine gastric tumors.	[11]
MKN-45, SNU-16, and U87-MG subcutaneous xenografts	MET	onartuzumab	^76^Br or ^89^Zr	^89^Zr-onartuzumab showed high gastric tumor uptake in mouse models.	[10]

Trastuzumab is FDA-approved in GC therapy. JS001 is in clinical trials for GC therapy (NCT02915432). AMG102 (Phase 3 RILOMET-1), onartuzumab (NCT01662869), and pertuzumab (JACOB) failed in clinical trials for GC therapy. MG7, D2101, H32 IgG, 75 IgG, 61 IgG, and mAb3481 used in the studies described in Table 1 were generated in the laboratory or purchased from commercial sources.

**Table 2:** Fluorescent-labeled antibodies used in molecular imaging of gastric tumors. Cetuximab failed as a first-line therapy in GC (EXPAND clinical trial). The anti-MG7 and anti-VEGF antibodies used in the studies [19,20] were produced in the laboratory or obtained from commercial sources.

Biological Model	Target	Antibody	Fluorescent Dye	Main Findings	Reference
MKN45 subcutaneous xenografts	EGFR	cetuximab	FITC	Higher fluorescence intensity was observed in cetuximab-FITC group than FITC-labelled isotype control.	[17]
BGC-823 and SGC-7901 subcutaneous xenografts	MG7	anti-MG7	Alexa Fluor 488	The strongest fluorescent signal was observed in the tumor at 48 h after intracardiac injection of AF488-labeled MG7 antibody.	[19]
APCmin mice with tumors in the small bowel and in the colon, SW480 and SW620 subcutaneous xenografts, and human tissues	VEGF	anti-VEGF	Alexa Fluor 488	VEGF specific signal was observed in tumors implanted in APCmin mice and transition zones between healthy and neoplastic tissue were identified based on VEGF expression patterns.	[20]
